# Recent Updates on Diabetes and Bone

**DOI:** 10.3390/ijms26178140

**Published:** 2025-08-22

**Authors:** Giacomina Brunetti

**Affiliations:** Department of Biosciences, Biotechnologies and Environment, University of Bari Aldo Moro, 70125 Bari, Italy; giacomina.brunetti@uniba.it

**Keywords:** diabetes, bone, Vitamin D, incretins, Glucagon-like peptide-2 (GLP-2), neurotensin, asprosin, irisin, TXNIP

## Abstract

Diabetes represents one of the major challenges in preserving health in the 21st century. It has been estimated that in 2050, 853 million subjects will live with diabetes. It was also reported that 3.4 million adults died from diabetes and related comorbidities. Chronic hyperglycemia, if not properly managed, leads to skeletal fragility with fracture risk that augments with age. In type 1 diabetes (T1D), the augmented fracture risk can be partially explained by lower areal bone mineral density (aBMD). Interestingly, in type 2 diabetes (T2D), the risk of fractures increases with normal or elevated aBMD. In this review, the recent updates on diabetes and bone health (2023–2025) are reported, thus describing bone quality and the role of mediators involved in diabetes pathogenesis. Consequently, the role of Vitamin D, Incretins, Glucagon-like peptide-2 (GLP-2), neurotensin, asprosin, irisin, and Thioredoxin-interacting protein (TXNIP) will be described considering the interplay between diabetes and bone health. The importance of monitoring diabetic patients’ bone health is underlined, together with the therapeutic approaches to avoid fractures.

## 1. Introduction

Diabetes represents one of the major challenges in preserving health in the 21st century. About 589 million adults are affected by this pathology in 2024, with 1.9 million pediatric subjects with type 1 diabetes (T1D). In these conditions in 2050, 853 million subjects will live with diabetes [1]. It was also reported that 3.4 million of adults died from diabetes and related comorbidities. The prevalence of diabetes among pregnant women is increasing, with an estimated ratio of about 1/5. Type 2 diabetes (T2D), underdiagnosed at about 43%, represents an alarming matter for diabetes diffusion and highlights the necessity to ameliorate prevention and optimize its care.

Diabetes is a chronic disease characterized by high glucose levels together with impairment of insulin levels/activity. Insulin is a hormone released by pancreatic β-cells, allowing the lowering of glucose levels in the bloodstream, thus leading all cells to metabolize or store it. Diabetes is also characterized by the altered levels of Haemoglobin A1c (HbA1c), which represent an indicator of glucose levels in the serum over a period of 90 days before the hematologic test. Thus, glucose and HbA1c levels are fundamental for diabetes diagnosis. If left untreated, the pathological increase in glucose levels affects the health of other tissues/organs, with the additional diagnosis of cardiovascular disease, neuropathy, nephropathy, retinopathy with vision loss, cognitive decline, cancer, liver disease, and bone disease. Different types of diabetes are known, and the pathogenesis is different. In detail, T1D is an autoimmune disease with immune system cells that damage β—pancreatic cells with consequent insulin deficiency. Both genetic and environmental factors can be involved in T1D development, and it can develop mostly in the pediatric population. T1D diagnosis is augmenting in high-income countries, maybe due to environmental changes. Untreated diabetes and its burden are increasing in low and middle-income countries [2]. T1D subjects need daily insulin injections to maintain glucose homeostasis and thus survive. Suboptimal glucose levels could lead to poor growth and vascular disease, and consistently, overall, in economically disadvantaged families, could cause complications with consequent disabilities.

T2D represents the most common type of diabetes, with about 90% of all patients worldwide. It is characterized by cell inability to respond to insulin, which is known as insulin resistance, and with time, leads to β-cell altered production of insulin. T2D is characterized by its silent upcoming and initial reversibility; therefore, by adjusting nutrition and physical activity, it can be reversed. It is most frequent in adults and is frequent in obese subjects. Pharmacological treatment includes glucosidase inhibitors, sulfonylureas, and glucagon-like peptide 1 receptor (GLP-1R), sometimes requiring insulin injections. Stem cell implantation, artificial pancreas, as well as pancreas transplantation, are also available as alternative approaches to counteract diabetes [3].

Monogenic diabetes can also be diagnosed following a genetic defect of β-cell function or insulin action [4]. A typical example of the former is represented by maturity-onset diabetes of the young (MODY), permanent neonatal diabetes (PNDM), and transient neonatal diabetes (TNDM). MODY appears before 25 years of age with a mutation in the glucokinase gene (GCK MODY) and hepato-nuclear factor gene (HNF1A MODY and HNF4A MODY). GCK MODY is characterized by lifelong mild fasting hyperglycemia, with rare microvascular complications. The most common HNF1A MODY is characterized by marked hyperglycemia and micro- and macrovascular complications. Differently, HNF4A MODY shows marked macrosomia and temporary neonatal hypoglycemia. TNDM is diagnosed in neonates under 6 months of age; the majority of them rescues, while others show mutations in the 6q24 region. Differently, half of PNDM patients displayed a mutation in genes encoding subunits of ATP-sensitive potassium channels. The other PNDM subjects showed heterozygous insulin gene mutations. Monogenic defects of insulin resistance can also be diagnosed and are characterized by the absence of obesity.

## 2. Methods

The review integrates the latest findings from 2023 to 2025 of diabetes-related bone disease, ensuring that readers are informed about the most current advances, such as high-resolution imaging studies (HR-pQCT), molecular insights into bone quality, and the role of different mediators. The search of papers was made using Pubmed and the keywords diabetes and bone combined with Vitamin D, Incretins, Glucagon-like peptide-2 (GLP-2), neurotensin, asprosin, irisin, and Thioredoxin-interacting protein (TXNIP). The originality of the keywords is evident because a review treating these molecules altogether is not present in Pubmed at the best of this author’s knowledge.

## 3. Skeletal Fragility in Diabetes

**Figure 1:**
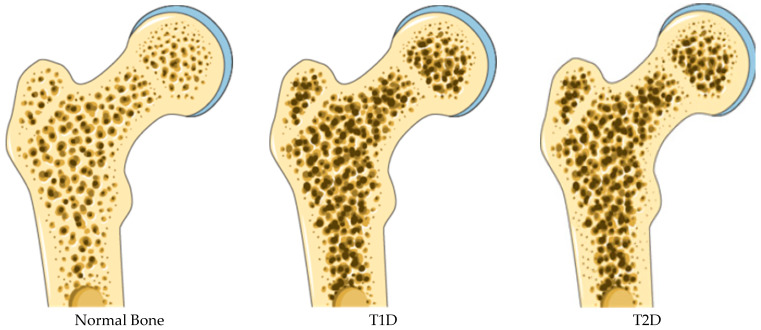
Cartoon depicting bone quality in diabetes, Adapted from SMART—**Servier Medical ART CC4 License**. BMD is generally decreased in T1D and normal or increased in T2D. Altered bone structure in T1D bone appears osteoporotic, whereas in T2D includes increased cortical porosity and reduced cortical density.

Chronic hyperglycemia, if not properly managed, leads to skeletal fragility with fracture risk that augments with age, in approximately 30% over 65 years old [5], Table 1 and Figure 1. T1D patients present low bone mineral density (BMD), whereas T2D patients show a smaller reduction in BMD compared to T1D patients; however, bone quality is low. Meta-analyses reported that T1D patients showed a 30–88% augmented fracture risk at different skeletal sites. In detail, it has been measured a 3.8–8.7-fold increase in hip fractures and 2.9 for vertebral fractures compared to non-diabetic patients [6]. The risk of fractures in T1D has also been reported for the pediatric age, even if its increased incidence compared with the healthy controls is linked to previous history of fractures, falls, medications, hypoglycemia, gender (males), and diabetes complications [7]. T2D patients showed 20–70% augmented risk of hip and nonvertebral fractures compared to normoglycemic subjects. The risk of developing fractures is linked to the duration or severity of diabetes; however, it is multifactorial and includes the augmented risk of falls due to hypoglycemic events [8]. In T1D, the augmented fracture risk can be partially sustained by lower areal bone mineral density (aBMD). Interestingly, in T2D, the risk of fractures increases with normal or elevated aBMD [6]. In T1D, femoral neck aBMD is mildly decreased, while the lumbar spine aBMD resulted in similar or slightly reduced with respect to the controls. T2D patients show normal-high aBMD at the hip, and the lumbar spine is also linked to high body weight, together with hyperinsulinemia [9]. Other authors evaluated the trabecular bone score (TBS) in diabetes, a simple method that estimates trabecular microstructure from DXA images [10,11]. In detail, in T1D, TBS resulted in a reduction, particularly in those with fractures. TBS resulted in a decrease in T2D patients, although it is lower in women than in men. The enhanced aBMD together with the reduced TBS is known as the Diabetes paradox. In pediatric age, TBS also resulted in damage from diabetes because the BMD peak was acquired between 0–20 years of life. Due to the knowledge that Dual-energy X-ray absorptiometry (DXA) evaluation can present some limits for a small body size, the use of quantitative computed tomography (QCT) has been tested [10]. In detail, in 118 young adult T1D patients, it has been reported that QCT shows a major sensitivity to measure BMD compared to DXA, in a major extent in patients without comorbidities. These uncomplicated patients also showed a reduced bone turnover and Bone Mass Index (BMI). The resulting early detection of bone complications led to the possibility of precocious therapy [10]. 3D studies allow for evaluation of volumetric BMD (vBMD) cortical and trabecular bone parameters.

In 2025, the Bone and Diabetes Working Group of the International Osteoporosis Foundation reports results arising from HR-pQCT studies on bone microarchitecture in diabetes [11]. T1D patients showed reduced trabecular bone number and density at the radius in adult and pediatric patients. The alteration of radio cortical bone was found in T1D patients with microvascular complications. Other studies demonstrated that tibial cortical thickness and BMD were reduced in T1D patients; however, trabecular bone was not affected. In 2025, Emerzian et al. reported that cortical bone material behavior in long-duration T1D femora from cadavers showed reduced post-yield toughness to fractures, minimal mineral crystallinity, elevated proline hydroxylation, and decreased glycosaminoglycan levels compared to the controls [12].

Using HR-pQCT or in vivo micro-indentation, it has been reported that T2D patients showed a bone quality deterioration with a deficit in bone material properties and an increased cortical porosity [9]. In 2025, it was found that HR-pQCT T2D patients showed reduced bone size compared to non-diabetic subjects. This alteration is associated with a lower tibial bone strength. Additionally, in long-lasting T2D (≥10 years), enhanced cortical porosity with reduced BMD was found to lead to a consequent increase in fracture risk, together with reduced trabecular thickness [13].

Hessellund et al. compared aBMD with vBMD, bone strength, cortical and trabecular parameters in T1D and T2D patients, showing in T1D reduced aBMD at femoral neck, higher vBMD, cortical vBMD, cortical area, and thickness together with reduced cortical porosity, increased stiffness, and failure load at tibia [14]. Differently in T2D, a higher aBMD was detected at the legs and arms, with elevated vBMD, cortical vBMD, cortical thickness and area, trabecular vBMD and number, bone stiffness, and failure load at the radius with respect to the controls [15].

Different authors evaluated the levels of both bone formation and resorption markers [16]. About the former, osteocalcin levels were reduced in T1D and T2D, and are inversely related to HbA1c [17,18]. About the latter, the levels of C-terminal cross-linked telopeptide (CTX) were consistently reduced with the low bone turnover in diabetes [19,20].

Maddaloni et al. evaluated bone fracture predictors in T2D patients enrolled in the EXenatide Study of Cardiovascular Event Lowering (EXSCEL) trial, reporting that the fractures are more evident in patients with diabetic neuropathy [21]. Additionally, optimal glycemic control in T1D pediatric subjects is strictly related to the right vascular parameters [22] and bone health in adults [23]. In detail, Gregory et al. in a cross-sectional study with long-term observation at radius and tibia involving 183 patients and 94 non-diabetic controls demonstrated that in T1D, suboptimal glycemic control, microvascular damage, advanced glycation end products (AGE) accumulation [24], and renal dysfunction are linked to altered trabecular and cortical bone. All these parameters can be reported to control levels by adjusting the glycemic control [23]. The role of microvascular disease leading to nephropathy, neuropathy, and retinopathy had unfavorable bone outcomes [25,26]. Different studies evaluated the mechanisms of bone disease in diabetes involving the key pathway of bone remodeling as the Receptor activator of nuclear factor kappa-Β (RANK)/Receptor activator of nuclear factor kappa-Β ligand (RANKL)/osteoprotegerin (OPG) pathway, WNT pathway [27], etc. In this review, the attention was focused on Vitamin D, Incretins, Glucagon-like peptide-2 (GLP-2), neurotensin, asprosin, irisin, and TXNIP.

## 4. Vitamin D and Diabetes

**Figure 2:**
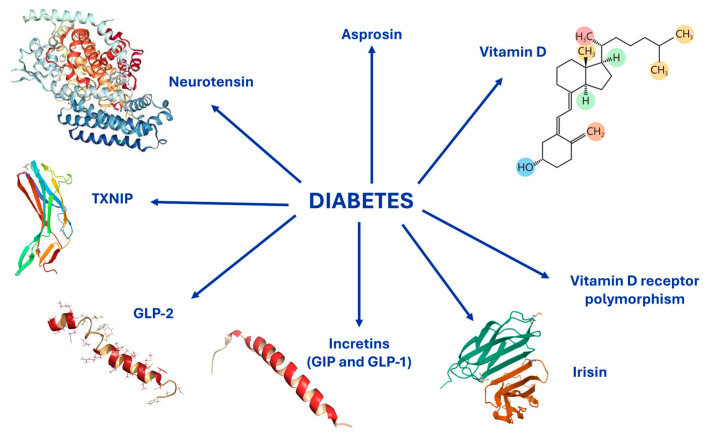
Mediators of bone disease in Diabetes: Vitamin D, Vitamin D receptor polymorphism, Incretins, Glucagon-like peptide-2 (GLP-2), neurotensin, asprosin, irisin, and Thioredoxin-interacting protein (TXNIP). All these molecules represent important therapeutic targets. Structures from PDB.

A clear interplay between bone and glucose metabolism has been reported to be linked to the fundamental role of Vitamin D in the management of bone diseases and/or diabetes (Figure 2). Vitamin D, a fat-soluble vitamin, exists in two different forms in our body: ergocalciferol (vitamin D2), produced by plants, and cholecalciferol (vitamin D3), produced in the skin as a consequence of exposure to ultraviolet radiation [28]. Vitamin D3 synthesis is profoundly linked to correct sunlight exposure, thus representing an essential nutrient that relies on environmental factors to maintain optimal concentration in the body [29]. It can be found as a natural or active form.

Upon meeting the circulation, vitamins D3 and D2 are metabolized in the liver, where the enzyme vitamin D-25-hydroxylase (CYP2R1) converts vitamins D3 and D2 into 25-hydroxyvitamin D (calcifediol). This intermediate form in the kidneys is additionally processed by the enzyme 25-hydroxyvitamin D-1α-hydroxylase (CYP27B1) to become the biologically active available form 1,25-dihydroxyvitamin D (calcitriol) [28].

1,25-dihydroxyvitamin D rapidly acts by binding the specific cytoplasmatic Vitamin D Receptor (VDR), broadly present in tissues and organs. 25-hydroxyvitamin D is 1000 times smaller in binding activity with respect to 1,25-dihydroxyvitamin D. The heterodimerization of VDR with the retinoid X receptor is fundamental to activate the transcription of numerous genes. VDR is part of the nuclear hormone receptor superfamily, and it is located on 12q13–12q14 chromosomes. Its polymorphism and ApaI, BsmI, FokI, and TaqI single-nucleotide polymorphism (SNP) can lead to altered Vitamin D activity and have been related to reduced BMD in T2D [30]. Vitamin D beneficial activities in diabetes are linked to its multiple effects, including detailed regulation of calcium and phosphorus homeostasis, anti-inflammatory, anti-angiogenic, antioxidant, and anti-proliferative actions. In different studies, it has been reported that optimizing simultaneously the Vitamin D levels and glycemic control can prevent comorbidities and maintain bone health [29,31,32]. Consistently, vitamin D intake significantly decreases T1D risk [33]. Vitamin D′s effect on immune cells is beneficial for autoimmune-damaged β-cells in T1D. In fact, different trials reported that calcitriol and insulin combined administration leads to beneficial effects for T1D management, due to the increase in the percentage of regulatory T cells. However, the use of only vitamin D is insufficient for diabetes management. Different considerations developed from studies on Vitamin D in T2D. In fact, it has been found that there is a reduced risk of T2D development through vitamin D administration of about −32% to 21% [34]. Other studies showed that Vitamin D administration was effective in pre-diabetic subjects with Vitamin D deficiency [35,36]. Furthermore, optimal levels of Vitamin D were necessary in diabetes to have a lower risk of venous thromboembolism [37].

Numerous cross-sectional and prospective observational studies demonstrated an inverse relationship between Vitamin D level and the prevalence or incidence of elevated HbA1c levels in T2D consistently; the supplementation with Vitamin D reduces HbA1c levels in T2D [38]. Vitamin D insufficiency determines oxidative stress and mitochondrial dysfunction, leading to impairment of insulin signaling, allowing T2D progression, which can be reversed by Vitamin D supplementation [28,38].

However, due to the key role of Vitamin D in diabetes and bone health, it is fundamental to ensure its optimal intake together with that of calcium (1000–1200 mg/day), mainly for patients at risk of fractures [39].

## 5. Incretins and Glucagon-like Peptide 2 (GLP-2)

Incretins are hormones produced by the gastrointestinal tract to modulate insulin levels in response to nutrient intake with a consequent effect on glucose circulating levels, however an effect on bone health was also found [40], Figure 2. Incretins include the Gastric Inhibitory Peptide (GIP) and Glucagon-like peptide 1 (GLP-1) [41]. The former is secreted by the enteroendocrine K cells of the small intestine inhibiting acid secretion in the stomach and stimulating insulin and glucagon synthesis according to glucose levels thus leading to the reduction in its circulating levels. The latter GLP-1 is released by enteroendocrine L cells in the small and large intestine and is quickly destroyed by dipeptidyl peptidase 4 (DPP-4). It promotes insulin release according to glucose levels and negatively affects stomach emptying. GIP actions are due to the binding with its receptor, a G protein-coupled receptor (GPCR) [42]. Following their interactions, a conformational change occurs, leading to a signaling cascade, with consequent increase in cyclic adenosine monophosphate (cAMP). The described conformational variation also determines the recruitment of β-arrestin. This step is essential to desensitize the receptor and hinders the intracellular signaling, as well as for receptor internalization. The GLP-1 receptor (GLP-1R) belongs to the class B1 GPCRs, which are activated by a two-step binding mechanism; however, the intracellular activation pathway is similar to GIPR.

Incretins, due to their mechanism of action, have been mimicked to realize new molecules that are primarily utilized for T2D treatment, but also in T1D patients even if they did not represent the standard care [14]. Interesting results have been obtained using the GLP-1 receptor agonists (GLP-1RAs), as dulaglutide and semaglutide. In vitro and in vivo animal model studies have reported that GLP-1RAs increase bone formation and resorption thus improving bone quality. Consistently, GLP-1RA treatment determines the levels of CTX, augments the amounts of amino-terminal pro-peptide of type I pro-collagen (PINP) and alkaline phosphatase (ALP). In osteoblasts, the bone-forming cells, GLP-1RAs increase RUNX2, β-catenin, PINP, and ALP. In rats, these molecules inhibit osteoclastogenesis. All these effects on bone cells lead to the reduction in BMD in T2D. GLP-1RAs (dulaglutide and semaglutide) affect adiponectin levels as demonstrated by a 12-month treatment in T2D patients [14]. This represents an important finding as adiponectin reduces oxidative stress and inflammation. Furthermore, a positive effect was also reported on myostatin, a cytokine that reduces the growth and development of muscle cells and increases osteoclastogenesis. In fact, its level decreased in T2D as a consequence of GLP1-RAs. Liraglutide, a representative GLP1-RA, is a new anti-diabetic and broadly used drug similar to the endogenous GLP-1 to increase insulin secretion. Thanks to the osteoblastic expression of receptors for GLP-1 in diabetes-related osteoporosis models, the continuous subcutaneous infusion of GLP-1 or Liraglutide normalized their impaired trabecular architecture and promoted bone formation [43].

A 2025 meta-analysis on anti-diabetic agent effectiveness on bone health in T2D patients reports that incretin-based therapy showed a better effect in preventing bone fractures than other medications [44]. Interestingly, GLP-1RAs appear to preserve periodontal and peri-implant health in T2D individuals than directly treating periodontitis or peri-implantitis [45]. It has been reported that GLP-1 RAs decreased the risk of fracture in T2D patients, and the beneficial outcome was related to the period of treatment [46].

At this time, the GLP-1RA Semaglutide is approved for T2D and obesity management, whereas its efficacy and safety in adults with T1D is ongoing, Clinicaltrials.gov number, NCT05537233 [47]. In this trial, in obese T1D adults, semaglutide treatment, with respect to automated insulin delivery use alone, significantly ameliorated HbA1c and glucose levels, together with body weight reduction. Semaglutide outdoes liraglutide in terms of both decrease in glycemia and weight loss [48]. In detail, an enhancement in vBMD at the tibia and radius was associated with the use of oral semaglutide in obese T2D, despite a significant body weight decrease. After 12 months of treatment, semaglutide determined TBS marginal increase, together with a modest, significant BMD decrease in the femora [14]. Using a T2D model treated with semaglutide and tirzepatide for 4 weeks or vehicle, it was found that glucose levels significantly decreased after treatment, although tirzepatide resulted in more weight loss compared with semaglutide [49]. The mean femur length was shorter in the tirzepatide group compared with controls. A cortical thickness reduction was measured in the semaglutide group compared to the control group. Interestingly, CTX and P1NP levels decreased as a consequence of the treatment. Semaglutide acted on *RANKL* and *OPG* mRNA levels, increasing the *OPG*/*RANKL* ratio. In vitro using bone marrow stromal cells, semaglutide sustains the proliferation and osteogenic differentiation of BMSCs *modulating* the Wnt/LRP5/β-catenin pathway [50]. Interestingly, it has been found in T2D patients undergoing posterior lumbar fusion surgery taking semaglutide preoperatively that consistent decreases in aggregated 90-day adverse events, with similar odds of hospital readmission [51].

Tirzepatide was studied in the SURMOUNT trials in individuals with overweight (BMI > 27 kg/m^2^) with or without T2D. Tirzepatide determined substantial weight loss of up to 20% in obese patients without T2D [52]. The majority of patients with obesity and prediabetes reversed glycemia. In the SURMOUNT-2 trial, the decrease in body weight in T2D patients was found [53]. Additionally, in the SURMOUNT-3 trial, tirzepatide provided substantial additional body loss in participants who had achieved ≥5.0% weight reduction with intensive lifestyle intervention [54]. Attractively, in the SURMOUNT-4 trial, a good percentage (46.2%) of tirzepatide-treated patients sustained a mean weight loss of at least 10% 1 year after discontinuation of tirzepatide [55].

Other GIP, GLP-1, and glucagon receptor agonists, such as Retatrutide, for T2D management and bone disease are currently under investigation, considering the positive effect on this pathology management [56].

GLP-2 is a 33-amino-acid peptide, secreted by gut L-cells, initially known to stimulate intestinal growth; it also increases BMD, calcium absorption, and decreases osteoclast activity in in vivo models [57]. In postmenopausal women, GLP-2 can suppress bone turnover and sustain bone growth. Its activity is the result of the direct interaction with GLP-2 receptor on osteoclasts as well as of its involvement in the gut- pancreas-bone axis through parathormone (PTH) (Figure 2). Subcutaneous GIP and GLP-2 reduced CTX and increased Procollagen type I N-terminal Propeptide (P1NP) in individuals with T2D [58].

## 6. Neuropeptides and Diabetes

Neuropeptides affect bone cell activity; among these, neurotensin, a 13-amino acid peptide, has an important role. This is a neurotransmitter released by the central nervous system and also by the enteroendocrine N cells of the small bowel, which regulates energy balance and inflammation [59]. Neurotensin stimulates lipid absorption in the gut in the presence of augmented absorption of lipids. Its precursor, proneurotensin, in high concentration is predictive of obesity, cardiovascular events, etc. Interestingly, a strong link has been demonstrated between proneurotensin levels and altered glucose/insulin metabolism. At the baseline, proneurotensin levels were not statistically different comparing overweight/obese and normal-weight children, whereas at 6.5 years follow-up, elevated proneurotensin levels are linked to the altered β-cell activity. Additionally, T2D and poor glucose control are characterized by high levels of proneurotensin. Interestingly, the increase in its levels are predictors of onset diabetes, overall, in women (Figure 2). In T2D, proneurotensin levels resulted in increased osteopenic/osteoporotic patients compared to T2D subjects with normal BMD. Its plasma levels are inversely related to BMD and T-score at total and neck femur, as well as worse TBS at lumbar spine. Proneurotensin levels also correlated with osteopontin, P1NP, Tumor Necrosis Factor alpha (TNFα), and Interleukin-1beta (IL-1β). It has also been reported that proneurotensin can predict T2D as well as its comorbidities [60].

## 7. Asprosin

Asprosin is a peptide hormone released from white adipose tissue in a fasting state and shows glucogenic and orexigenic effects. Asprosin is linked to metabolic syndrome, glucose metabolism, insulin resistance, and lipid profiling. Altered asprosin levels were detected in different diseases, including T2D [61]. It has diagnostic and therapeutic roles, because it is related to T2D complications, including the bone disease, Figure 2 [61]. Consistently, it has been shown by Roomi et al. that serum asprosin levels are enhanced in postmenopausal T2D women with osteoporosis with respect to postmenopausal T2D women without osteoporosis and controls [61]. The same authors reported a significant correlation between osteoporosis radiological indicators and osteocalcin in T2D women. Moreover, they reported that in T2D women, asprosin levels were positively related to CTX amounts and glycemic markers. Furthermore, they showed that the Receiver operator characteristic (ROC) curve defined the asprosin cutoff value discriminating osteoporosis T2D postmenopausal women from non-osteoporosis T2D postmenopausal women at 90% sensitivity. Additionally, Song et al. explored the relationship between serum asprosin levels, microRNA-21 (miR-21) expression, and delayed healing after surgery in patients with osteoporotic vertebral compression fractures, showing that asprosin amounts were increased, whereas miR-21 levels were reduced in patients with delayed healing after surgery, also characterized by a major prevalence of diabetes [62].

## 8. Irisin

Irisin is an adipo-myokine hormone produced during exercise, affecting bone remodeling and showing therapeutic potential for diseases as metabolic disorders, sarcopenia, osteoporosis, diabetes, obesity, and neurodegenerative diseases, including Alzheimer’s disease [63]. The most recent paper on irisin and diabetes focused on mechanisms characterizing bone disease (Figure 2). Using a diabetic mouse model induced by intraperitoneal streptozotocin injection, irisin treatment suppressed ferroptosis and improved bone loss, as demonstrated by reduced iron overload and lipid peroxidation, augmented antioxidant capability, and inhibition of the ferroptosis pathway in bone tissues [64]. Furthermore, the same authors using in vitro studies showed that irisin overexpression significantly ameliorated high glucose-induced ferroptosis and stimulated osteogenesis. Consistently, irisin overexpression softened ferroptosis in osteoblasts through the inhibition of the eukaryotic initiation factor 2 alpha (eIF2α)/activated transcription factor 4 (ATF4)/C/EBP-homologous protein (CHOP) pathway. Using the same in vivo model, Mohsin et al. reported that T1D deteriorates the trabecular bone microstructure by augmenting trabecular separation (Tb-Sp) and reducing bone volume fraction (BV/TV), trabecular thickness (Tb-Th), and BMD [65]. Irisin treatment improved bone quality by enhancing trabecular number and BV/TV, BMD, and Tb-Sp. The bone microarchitecture deterioration in this diabetes model is mainly due to reduced bone formation associated with minimal osteocalcin and elevated sclerostin levels in bone samples. Consistently, the irisin treatment significantly decreased the serum and bone sclerostin levels, enhanced the serum CTX1 levels, whereas a non-significant improvement in osteocalcin levels was reported. Moreover, Hou et al. evaluated the role of irisin using the same diabetic model and T2D patients’ samples, reporting that with respect to healthy subjects, it was evident that the reduced serum irisin levels and BMD of L1-L4 lumbar spine, total hip, femoral neck, and Wards in postmenopausal T2D patients, further diminished in osteoporosis T2D patients [66]. Furthermore, the same authors showed that recombinant Irisin injection ameliorated diabetic bone loss and inflammation in diabetic mice.

## 9. Thioredoxin-Interacting Protein (TXNIP)

TXNIP has been principally described as an endogenous negative modulator of TXN action in oxidative phosphorylation. TXNIP involvement has been demonstrated in cell inflammation, immunity, and glucolipid metabolism. Thus, it is involved in chronic inflammatory and metabolic diseases, including cardiovascular diseases, hepatic lipidosis, and diabetes, Figure 2 [67]. Further insights demonstrated TXNIP involvement in bone remodeling, modulating the crosstalk between osteoblasts and osteoclasts. In detail, TXNIP modulates Tnfsf11 transcription and RANKL expression via the novel transcription-related Ecdysoneless (Ecd)-P300 axis. *TXNIP*^−/−^ mice are helpful for the management of diabetic-related osteoporosis. Thus, targeting TXNIP is useful to prevent bone loss and as an alternative to RANKL inhibition. Additionally, TXNIP has been described as an intracellular mediator of the anti-proliferative effects of extracellular high glucose levels on osteoblasts [68]. In addition, it has been reported that the Forkhead box protein O1 (FOXO1)/TXNIP pathway was identified as a key target in T2D bone disease [69].

## 10. Bone Health Monitoring and Cure in Diabetes

Bone disease represents a common comorbidity of diabetes. The American Diabetes Association, as well as all different diabetes and bone societies worldwide, dictate a number of recommendations to preserve bone health in diabetes [39,70]. It is important to evaluate and continuously monitor the fracture risk overall in older adults. Monitor BMD using DXA both in adults and younger patients every 2–3 years. It is fundamental to know bone status in the patients at the moment to choose the pharmacological approach, thus avoiding drugs with a potential high effect on fracture risk is recommended. The use of glucose-lowering drugs with few hypoglycemic events should be preferred to have a low risk of falls, that are dangerous for the skeleton. The recommended age-specific right intake of calcium and Vitamin D should be indicated with also attention to their diet. In detail, daily allowance is 600IU for people aged 51–70 years and 800 IU for those <70 years. Antiresorptive and/or osteoanabolic drugs should be recommended for diabetic patients at high risk of fracture, low BMD (T-score ≤ −2) as well as history of fragility fracture. As anti-osteoporotic drugs reducing bone resorption bisphosphonates, denosumab, and selective estrogen receptor modulators can be useful. Differently, as osteoanabolic agents teriparatide, abaloparatide, and romosozumab are recommended. For primary prevention of fragility fracture in diabetes Denosumab is preferred but keeping attention at glomerular filtration rate. Anti-osteoporotic treatment is fundamental for secondary prevention of fragility fractures. Prasad et al. reported that in T2D the use of bisphosphonate Zoledronate, denosumab, and teriparatide decreases the risk of falls with consequent reduced risk of fractures [71].

## 11. Conclusions

Bone health is affected by diabetes through different mechanisms, among others, and particular attention should be kept towards VDR polymorphisms, Vitamin D, incretins, GLP-2, neurotensin, asprosin, irisin, and TXNIP levels. Appropriate medications aiming to maintain optimal glucose circulating levels are useful to avoid comorbidities, including bone disease. However, this requires continuous monitoring and appropriate care. Additionally, the identification of new diabetes mediators could be useful for its prevention and management.

## Figures and Tables

**Table 1:** Bone characteristics in Diabetes.

	T1D	T2D
aBMD	low	Normal/elevated
TBS	decreased	decreased
Trabecular Number	reduced	
Cortical Bone	Reduced Cortical Thickness	Increased Cortical Porosity
CTX	Reduced levels	Reduced levels
TRACP-5b	Increased levels	Reduced levels
P1NP	Reduced levels	Reduced levels
Bone-ALP	Reduced levels	Reduced levels
Osteocalcin	Reduced levels	Reduced levels
